# Can Nutritional Supplements Benefit Patients With Nonalcoholic Steatohepatitis and Nonalcoholic Fatty Liver Disease?

**DOI:** 10.7759/cureus.40849

**Published:** 2023-06-23

**Authors:** Ahmed M Baradeiya, Khaled M Taghlabi, Abdelhalim N Saleh, Sindhura Manikonda, Siffat S Salim

**Affiliations:** 1 Advanced Liver Therapies Research, Baylor College of Medicine, Houston, USA; 2 Neurological Surgery, Houston Methodist Hospital, Houston, USA; 3 Nephrology, Greater Houston Kidney Specialist, Houston, USA; 4 Internal Medicine, Shadan Institute of Medical Sciences, Hyderabad, IND; 5 Surgery, Holy Family Red Crescent Medical College Hospital, Dhaka, BGD

**Keywords:** risk factors, epidemiology, supplement use in nash, nash, nafld

## Abstract

A characteristic of nonalcoholic fatty liver disease (NAFLD) is the buildup of excess fat in the liver which encompasses various clinical phases, including steatosis, inflammation, ballooning, fibrosis, and liver cirrhosis. Nonalcoholic steatohepatitis (NASH) represents a severe form of NAFLD. The prevalence of NAFLD, particularly NASH, is notably high among Hispanics and those with morbid obesity. Diabetes, obesity, and dyslipidemia are significant risk factors in patients with NAFLD. The pathogenesis of NAFLD involves complex interactions between hormonal, nutritional, and genetic factors. Different clinical trials have been conducted to determine if there are any supplements that could help patients with NASH. Evidence has shown that vitamin E decreased the NAFLD activity score but not fibrosis. Our review summarizes the influence of supplementation on patients with NAFLD and NASH, focusing on the use of different clinical trials, systematic reviews, and meta-analyses. In the future, patients and physicians will play crucial roles in exploring diverse approaches and finding effective solutions to address this growing issue.

## Introduction and background

Nonalcoholic fatty liver disease (NAFLD) is a condition in which excess fat is stored in the liver [1]. The liver stores carbohydrates in the form of glycogen [1]. Healthy human liver cells contain little or no fat; however, the presence of more than 5% droplet of fat in the liver is considered pathological or abnormal [1]. Nonalcoholic steatohepatitis (NASH) is a clinically serious form of NAFLD characterized by excess accumulation of triglycerides (steatosis) [2]. NAFLD is currently recognized as one of the most chronic liver diseases and globally affects approximately 25% of the adult population [2]. The following four different clinical phases have been recognized for NAFLD: phase one is characterized by steatosis; phase two histologically presents with inflammation and ballooning and is sometimes associated with increased inflammatory markers; phase three is defined as the presence of NASH with persistent inflammation that leads to scarring (liver fibrosis); and phase four leads to more serious conditions such as liver cirrhosis [3]. The disease affects 90% of morbidly obese individuals and up to 70% of overweight individuals [4]. NAFLD and NASH have been observed in higher percentages in Hispanics, intermediate in whites, and lowest in Black people [4]. Asians tend to have more lobular inflammation and higher grades of ballooning than other ethnicities [4]. The disease involves complex interactions among host genetics, gut microbiota, and environmental factors [5]. Innate immune activation and inflammation are two of the most characteristic features of NASH, including the sterile programmed cell death process as a result of lipotoxicity in the hepatocytes, as well as altered liver-gut axis function involving translocation of the bacteria into the portal circulation [5].

Obesity and related ailments, such as cardiovascular diseases and type 2 diabetes mellitus (T2DM), have been linked to oxidative stress, which has been recognized as a major contributing factor in NAFLD [6]. Through the nuclear factor kappa B (NF-kB) pathway, consuming a lot of glucose and omega-6 polyunsaturated fatty acids (n-6 PUFAs) causes inflammation through the NF-kB light-chain enhancer of activated B cell-mediated route [6]. Omega 3 polyunsaturated fatty acids (n-3 PUFAs) can be beneficial against metabolic and cardiovascular disorders due to their antioxidant and anti-inflammatory effects [6]. The n-6 PUFA/n-3 PUFA ratio is significantly higher in the livers of NAFLD patients, which may favor lipid synthesis over oxidation and cause steatosis [6]. Consumption of vitamins and minerals, such as n-3 PUFA, vitamin A, vitamin E, vitamin C, selenium, and alpha-lipoic acid, acts as an antioxidant that benefits NAFLD patients [6]. Multiple observational studies in different populations have suggested that coffee consumption is associated with a reduced risk of NAFLD [7]. Caffeine prevents or reverses hepatic fibrosis through several mechanisms, including acting as an A2A receptor antagonist that affects the activation of hepatic stellate cells, which present lipid antigens to natural killer T cells [7]. Stx17 is a protein involved in autophagosome/lysosome fusion and is ubiquitinated and downregulated in NASH. Dietary B12 and folic acid supplementation increases Stx17 expression, increases autophagy, and slows NASH progression [8]. Extra virgin olive oil is a functional food with high levels of monosaturated fatty acids that reduce fat accumulation in the liver [9]. Nitro fatty acid formation has been observed in mice after digestion with extra virgin olive oil [9]. Multiple studies have found that nitro fatty acids protect against NAFLD by acting as anti-inflammatory and antioxidant agents [9].

## Review

Search strategy

Using NAFLD, NASH, supplement use in NASH and NAFLD, epidemiology, risk factors, and pathophysiology of NASH and NAFLD as research keywords, articles were reviewed to illustrate and analyze the role of supplements in the improvement of NAFLD, NASH, and liver function tests. Studies published between 2011 and 2023 were considered for inclusion in this review. Medline was used as the primary database. Data were collected from literature reviews, systematic reviews, meta-analyses, and several types of clinical trials, cohort studies, and retrospective studies. There were no age or ethnicity limitations to the search. Only articles published in the English language were included in this review.

This section will discuss the epidemiology, risk factors, and pathophysiology of NAFLD and NASH; the role of multiple kinds of supplements in both diseases; and how supplements can benefit NASH patients and improve their liver function tests.

Epidemiology of NAFLD

The incidence and prevalence of NAFLD are increasing quickly around the globe, ranging from 13% in Africa between 1989 and 2015 to 40% in Southeast Asia between 1999 and 2019 [10]. The prevalence is projected to increase by up to 56% between 2016 and 2030 in China, Japan, Germany, France, Italy, Spain, the United Kingdom, and the United States [10]. NASH was the fastest-growing cause of liver cancer death globally, especially in America, from 2010 to 2019, driven by the rapidly rising obesity rate [11]. An incidence of 31 per 1,000 people per year was found in a Japanese study that examined raised aminotransferase levels, weight gain, and the development of insulin resistance over five years to categorize patients with NAFLD [12]. Later, a retrospective investigation performed in England revealed a significantly lower incidence of 29 per 10,000 people annually [12]. An extensive meta-analysis described the pooled regional incidence of NAFLD in Asia to be 28 per 1,000 persons per year [12]. Table 1 shows the prevalence of NAFLD in the different regions and countries.

**Table 1:** Prevalence of NAFLD in relation to specific regions. NAFLD: nonalcoholic fatty live disease

Region/Country	Prevalence of NAFLD (%)
United States	25%
Africa	13%
Southeast Asia	40%
South Korea	25%
India	17%
Middle East	30%

Risk factors of NAFLD

Diabetes and obesity are major risk factors in patients with NAFLD [10]. T2DM has a strong relationship with the progression of NAFLD; in fact, more than 50% of patients with T2DM have NAFLD [13]. Body mass index (BMI) and waist circumference are positively correlated with NAFLD [13]. Metabolic syndrome (MS) has multiple manifestations that increase waist circumference, hyperglycemia, dyslipidemia, and systemic hypertension [13]. The incidence of MS has increased in recent years, similar to NAFLD, and both diseases are closely linked [13]. Recent studies have shown that age, smoking, and gut microbiota directly affect NAFLD and its progression to NASH [13]. Gut barrier disruption leads to the translocation of overgrowing bacteria to the mucosa and circulation and enhances liver inflammation [14]. Increased bacterial translocation from the gut to the blood leads to continuous metabolic bacteremia [14]. The most extensively studied microbial molecule is lipopolysaccharide (LPS), a cell wall component of gram-negative bacteria [14]. Multiple human and animal studies have shown that systemic LPS concentration is significantly elevated in NAFLD [14]. Table 2 shows the prevalence of NAFLD in association with other significant risk factors.

**Table 2:** Prevalence of NAFLD in relation to other risk factors. T2DM: type 2 diabetes mellitus; HTN: hypertension; NAFLD: nonalcoholic fatty live disease

Risk factors	NAFLD (%)
T2DM	50%
Severely obese	90%
HTN	40%
Dyslipidemia	72.1%
Age 40–49 years (male)	45%
Age >70 years (male)	25%
Age 60–69 years (female)	31%
Age >70 years (female)	20%

Pathophysiology of NAFLD and NASH

**Figure 1:**
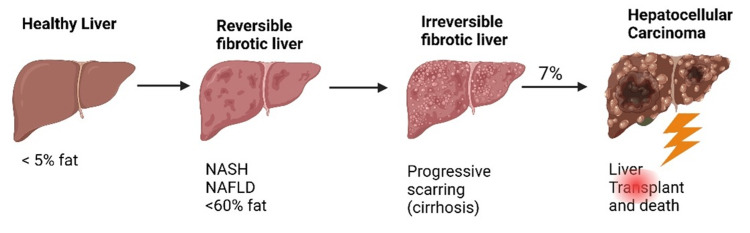
Pathological stages of NAFLD and NASH. The figure is created by author Baradeiya AM in BioRender.com and reprinted with permission [19,20]. NAFLD: nonalcoholic fatty live disease; NASH: nonalcoholic steatohepatitis

The pathogenesis of NAFLD and NASH is multifactorial, and many mechanisms have been proposed as possible causes of fatty liver infiltration [17]. In recent years, many animal studies have investigated the pathophysiology of NAFLD and NASH in dietary models that are high in fructose and high-fat or choline-deficient diets. Various cells of the body, including liver cells, create cell signaling chemicals called cytokines. They have a crucial role as inflammatory disease mediators in high uric acid environments. They comprise chemokines, interleukins (IL), transforming growth factors (TGFs), tumor necrosis factors, and interferons. Inflammation and lipid buildup are important factors in NAFLD [2,18]. Based on this evidence, it has been suggested that the development of NASH due to fat deposition in the liver increases insulin resistance and causes cellular and molecular changes involving oxidative stress [18]. Hormonal, nutritional, and genetic factors are intricately connected to NAFLD [19]. The systemic decrease of gut-derived hormones that promote satiety (e.g., glucagon-like peptide 1) and an increase in gut-derived hormones that stimulate hunger (e.g., ghrelin), which are linked to an increase in circulating triglyceride levels and are therefore implicated in NAFLD, occur in response to certain macronutrients, such as high fat and sugar [19]. Growth of insulin resistance results in increased adipocyte lipolysis, high free fatty acids, and increased gluconeogenesis, which lead to more lipids in the liver and accentuated triglycerides in the form of low-density lipoproteins [19]. A genome-wide association study on NAFLD and NASH by Anstee et al. [20] compared the genetic profiles of those with fatty liver disease to those seen in the general population. The study reported that fatty liver disease had a varying frequency of sequences in four different regions of the human genome [20]. Figure 1 illustrates the pathological stages of NAFLD and NASH.

Role of supplements in NAFLD and NASH

Vitamin E is a naturally occurring fat-soluble vitamin with the reputation of being a crucial antioxidant [21]. The PIVENS trial by Sanyal et al. [21] reassessed the associations between changes in alanine transaminase (ALT) levels in a group of subjects taking pioglitazone versus vitamin E versus Placebo in NASH. The study found that ALT improvement was greater (48%) in vitamin E receivers compared to placebo recipients (16%) (p < 0.001). ALT responses among those who received vitamin E were linked to lower NAFLD activity scores (NAS) (p < 0.001) but not fibrosis scores (p = 0.34) [21]. Silymarin, an extract of milk thistle (*Silybum marianum*), can mitigate lipid peroxidation and free radical injury [22]. Five medical facilities in the United States participated in a multicenter, phase II, randomized, double-blind, placebo-controlled trial using silymarin. For 48 weeks, patients were randomly assigned to receive a placebo, silymarin 420 mg, or silymarin 700 mg. For the participants with NAFLD who also had NASH but no cirrhosis and an activity score (NAS) of 4, the primary endpoint of the research was a histological improvement of 2 points in NASH; however, there was no statistically significant improvement in fibrosis [22]. Curcumin, a yellow pigment isolated from *Curcuma longa Linn*, has reportedly been effective in reducing oxidative stress and inflammatory cascades [23]. In a placebo-controlled, double-blinded, randomized clinical trial conducted in Iran, eligible patients who satisfied the inclusion criteria were randomly assigned to receive three doses of a matched placebo or 500 mg of curcumin [23]. Hepatic fibrosis was significantly reduced by curcumin administration (p < 0.001) [23]. Omega-3 fatty acids (O3-FAs) reduced serum triglyceride [24]. A meta-analysis of human interventional studies showed that O3-FAs decreased liver fat content in the absence of weight loss [24]. In a 12-week, multicenter, randomized, placebo-controlled, double-blinded study conducted in Sweden, 200 mg fenofibrate, 14 g omega-3 carboxylic (OM-3CA), or matching placebos were administered to patients with hypertriglyceridemia and fatty liver. The study reported out serum triglyceride decreased with OM-3CA (p = 0.02) and fenofibrate (p < 0.001) but not fat in the liver [24]. Improvement and even protection from the progression of NAFLD have been documented for the ketogenic diet. These findings collectively imply that the most effective treatment for NAFLD to date involves dietary and exercise-based lifestyle adjustments that may reverse NAFLD [13].

Synbiotics are formulas containing probiotics plus prebiotics [25]. A probiotic is a microbe that, when provided in a suitable amount, provides positive characteristics for the host, and a prebiotic is a non-digestible carbohydrate that affects the host by selectively activating the beneficial bacteria in the colon [25]. At Hospital Das Clinicas, Universidade Federal de Minas Gerais, Belo Horizonte, Brazil, 50 patients with NASH participated in a controlled clinical trial. The patients were randomly divided into two groups, namely, those who received a synbiotic (n = 27) and those who did not (n = 23), along with lifestyle modifications [25]. In the treatment of NASH, synbiotic supplementation was superior to lifestyle modification alone compared to lifestyle change alone [25]. Evidence indicates that brown algae polysaccharides are dietary fibers that can modulate energy intake, counteract obesity, exert an anti-steatotic effect, and prevent NAFLD [26]. Recent preclinical research has shown that brown seaweed can lower MS risk factors [27]. In a study, rats with NAFLD and rats with NASH were used as animal models for liver steatosis to examine the beneficial effects of formulations containing a phytocomplex from seaweed and chromium picolinate. These formulations significantly reduced hepatic fat deposition in both models and plasma cytokines such as interleukin 6, tumor necrosis factor, and C-reactive protein [27]. Table 3 shows the role of supplements reported in systematic reviews and meta-analyses.

**Table 3:** Role of supplements in NASH and NAFLD from systematic reviews and meta-analyses. NAFLD: nonalcoholic fatty live disease; NASH: nonalcoholic steatohepatitis; BMI: body mass index; ALT: alanine transaminase; AST: aspartate transaminase; TG: triglyceride; TC: total cholesterol; LDL-C: low-density lipoprotein-cholesterol; MS: metabolic syndrome; RCCT: randomized control clinical trials

Author (year)	Study design	Supplements		Conclusion
Carpi et al. (2022) [28]	Systematic review	Probiotics		Probiotics do not play a healing role and work by preventing the formation of toxic metabolites in the liver
Yang et al. (2022) [29]	Systematic review and meta-analysis	Polyphenols	Curcumin	Decreased BMI, ALT, AST, TG, and TC
			Naringenin	Decreased percentage of NAFLD, TG, TC, and LDL-C
			Silymarin	Improved AST, ALT, liver fat, and stiffness, which play a liver-protective role
Gurusamy et al. (2018) [30]	Meta-analysis and review of 202 RCTs	Nutritional supplementation		The evidence indicates considerable uncertainty about effect for people with NAFLD
Hariri et al. (2019) [31]	Systematic review of RCCTs	Vitamin D		Vitamin D improves lipid profile and inflammatory mediators without significant effect on liver enzymes and might improve symptoms of NAFLD
Sharpton et al. (2019) [32]	Systematic review and meta-analyses	Probiotics and synbiotics		Improvement of liver-specific markers (ALT), liver stiffness measurement by elastography, and liver steatosis
Pani et al. (2020) [33]	Systematic review	Inositol deficiencies		Increased fatty liver in animals
Wei et al. (2021) [34]	Systematic review and meta-analyses	Resveratrol		Resveratrol supplementation did not result in significant changes in ALT and AST
Dludla et al. (2020) [35]	Meta-analyses of RCT	Coenzyme Q_10_		Lower inflammation markers in MS patients
Lee et al. (2020) [36]	Systematic review and meta-analyses	Omega-3 polyunsaturated fatty acids		Significantly improved TG, HDL, TC, and BMI
Liu et al. (2023) [37]	Systematic review and meta-analyses	L-carnitine		Improves liver function tests and regulates TG
Chung et al. (2014) [38]	Systematic review and meta-analyses	High-fructose corn syrup		There was insufficient evidence to draw conclusions

## Conclusions

NASH is rapidly rising worldwide and is the fastest-growing cause of liver cancer death globally, especially in the Spanish community. Lifestyle plays a significant role according to new data. Therefore, it is important to study the role of supplements and how they benefit patients with NAFLD and NASH. According to numerous studies published in the last 10 years, supplementation plays a role in helping NAFLD and NASH patients by improving liver function tests and other factors; however, no significant improvement has been reported in the level of fat in the liver or improvement in any scarring tissue. Both physicians and patients should be educated about the disease and lifestyle changes, encouraging patients to participate in ongoing pharmaceutical clinical trials worldwide to help find a new solution.
